# Mn-doped Ge and Si: A Review of the Experimental Status

**DOI:** 10.3390/ma3125054

**Published:** 2010-11-26

**Authors:** Shengqiang Zhou, Heidemarie Schmidt

**Affiliations:** 1 Institute of Ion Beam Physics and Materials Research, Forschungszentrum Dresden-Rossendorf, P.O. Box 510119, 01314 Dresden, Germany; E-Mail: heidemarie.schmidt@fzd.de; 2 State Key Laboratory of Nuclear Physics and Technology, School of Physics, Peking University, Beijing 100871, China

**Keywords:** diluted ferromagnetic semiconductor

## Abstract

Diluted ferromagnetic semiconductors (FMS) are in the focus of intense research due to their potential applications in spintronics and their striking new physical properties. So far Mn-doped III-V compound semiconductors such as GaMnAs are the most important and best understood ones, but they are ferromagnetic only at well below room temperature. An interesting alternative could be magnetic semiconductors based on elemental semiconductors, also owing to their compatibility with Si microelectronics. In the last decades, considerable amount of work has been devoted to fabricate Mn-doped Ge and Si FMS. In this article, the structural, magnetic and magneto-transport properties of Mn-doped Ge and Si will be reviewed.

## 1. Background and Introduction

### 1.1. GaMnAs: Promises and Questions

In traditional electronic devices, charge and spin are used separately. Charge, on one hand, is used for the computing. Transistors operate by controlling the flow of charge carriers through the semiconductor by applied electric fields. Spin, on the other hand, is used for magnetic data storage. The word “spintronics” (short for “spin electronics”) refers to devices that manipulate the spin degree of freedom. A new generation of devices based on the manipulation of spins may have completely new functionality, therefore could drastically improve the computational speed and reduce power consumption. The first successful application of spintronics is the Giant Magnetoresistive (GMR) spin-valve read-head for magnetic hard-disk drives [1,2]. Magnetoresistance describes the dependence of electric resistance on the magnetic field applied to the material. Datta and Das [3] extended the principle of spintronics to semiconductors. They proposed a spin-FET (field effect transistor), where the source and the drain are ferromagnets acting as the injector and detector of the electron spin. By modifying the gate voltage, the charge carrier spin can be controlled. The spin injector can be a ferromagnetic metal or a ferromagnetic semiconductor. The crucial problem is the efficiency of the spin injection, i.e., the amount of carriers that can keep their spin state while moving a long enough distance. While the degree of spin polarization for spin injection from a ferromagnetic metal to a semiconductor is limited due to the conductivity mismatch [4], a ferromagnetic semiconductor could allow a robust spin injection into a nonmagnetic semiconductor. The diluted ferromagnetic semiconductor (FMS) GaMnAs is most well understood and promising for application in spintronics. The main obstacle is that the highest Curie temperature (*T_C_*) of GaMnAs is reported to be 173 K [5], which is far below room temperature. Nevertheless, spin-involved devices based on GaMnAs, namely a spin-polarized light emitter [6], a spin FET [7] and a spin valve [8], have been demonstrated at low temperature. Now it is well accepted that GaMnAs can be used as a test bed for future spintronics devices [9]. However, concerning the physical understanding of GaMnAs there are mainly three questions.

(1) The magnetic coupling and transport mechanism

Within the frame of the RKKY-Zener model [10], the ferromagnetic interaction between the local moments provided by substitutional Mn (Mn*_Ga_*) is mediated by the holes generated also by Mn*_Ga_*. For a low Mn concentration the Fermi energy lies in a Mn-induced impurity band and and the hole states are localized. However, the nature of the states at a higher hole concentration, responsible for high T*_C_*, remains controversial. Indeed even for samples with large Mn concentrations, more and more evidences indicate the impurity-band formation [11]. It is currently unclear whether the hole states close to the Fermi energy remain localized or become extended [12].

(2) New physics arising concerning GaMnAs FMS

The discovery of the GaMnAs FMS opened new routes to the successful combination of magnetism and semiconductor physics. Some phenomena, e.g., giant planar Hall effect [13], tunnelling anisotropic magnetoresistance [14], intrinsic anomalous Hall effect [15], which are usually very weak or not present in traditional semiconductors or ferromagnetic metals, have been observed.

(3) The low mobility

Besides the low T*_C_*, another obstacle is easily ignored, *i.e.* the low mobility. The hole mobility in GaMnAs with high T*_C_* is usually as low as 1–5 cm^2^/Vs. For usually doped unmagnetic GaAs with a comparable hole concentration, the hole mobility is in the range of 30–50 cm^2^/Vs (see the database of New Semiconductor Materials at http://www.ioffe.ru/SVA/NSM/). The hole mobility in GaMnAs might be physically limited by the large effective mass of holes localized inside an impurity band [16].

### 1.2. Alternatives: Mn-Doped Si and Ge

Note that the family of III-Mn-V compound semiconductors is the most successful FMS. Elemental semiconductors (Si and Ge) showing FMS characteristics would shed light on the physical understanding of FMS and might overcome the application obstacles in III-Mn-V compound semiconductor FMS. The relatively simple structure of Ge compared with compound semiconductors will make theoretical modelling easier. Especially, as a Si-technology compatible material, Ge-based FMS is more promising for industry applications.

The review focuses on the experimental work of Mn-doped Ge and Si. We mainly discuss the structure, magnetic and magneto-transport properties, to address the following questions:

- Is there any experimental proof that Mn can substitute Si or Ge?
- Are some Mn-rich precipitates formed in Si or Ge upon Mn doping?
- How can Mn-rich precipitates be avoided?
- Are there any magneto-transport experiments supporting carrier-mediated ferromagnetism in Mn-doped Si or Ge?

The heterostructures of Mn-silicides/Si and Mn-germinides/Ge(Si), e.g., MnSi/Si [17], Mn_5_Ge_3_/Ge [18], will not be discussed in this review.

## 2. Mn-Doped Ge

### 2.1. Substitutional Mn in Ge

Similar to Mn in GaAs, the solid solubility of Mn in Ge is as low as 10^15^ cm^−3^ under equilibrium conditions [19]. Woodbury and Tyler investigated the electrical properties of Mn-doped Ge. The temperature dependence of the electrical resistivity and Hall coefficient indicate that Mn introduces two acceptor levels in Ge at 0.16 ± 0.01 eV from the valence band and 0.37 ± 0.02 eV from the conduction band. In order to fabricate Ge:Mn FMS, non-equilibrium preparation methods, like low-temperature molecular beam epitaxy (LT-MBE) and ion implantation, were applied. The introduced Mn concentration introduced amounts to percentage. Indeed, a hole concentration ranging from 10^17^–10^20^ cm^−3^ has been reported by various groups [20,21,22,23,24,25]. Complementarily, X-ray absorption (XA) spectroscopy has been used to probe the local electronic structure and the charge state of the Mn impurities in the Ge matrix. Various investigations confirm that in Ge the diluted Mn ions are in the 2+ ionic states and substitute the Ge sites [26,27,28,29]. Li *et al*. [21] performed a quantitative analysis of the lattice location of Mn in Ge. They observed that between 20–30% Mn ions are in the substitutional sites and a post-annealing at low temperatures increases this fraction to 40–50%. Some interstitial Mn have been converted to substitutional Mn during the annealing process.

**Figure 1:**
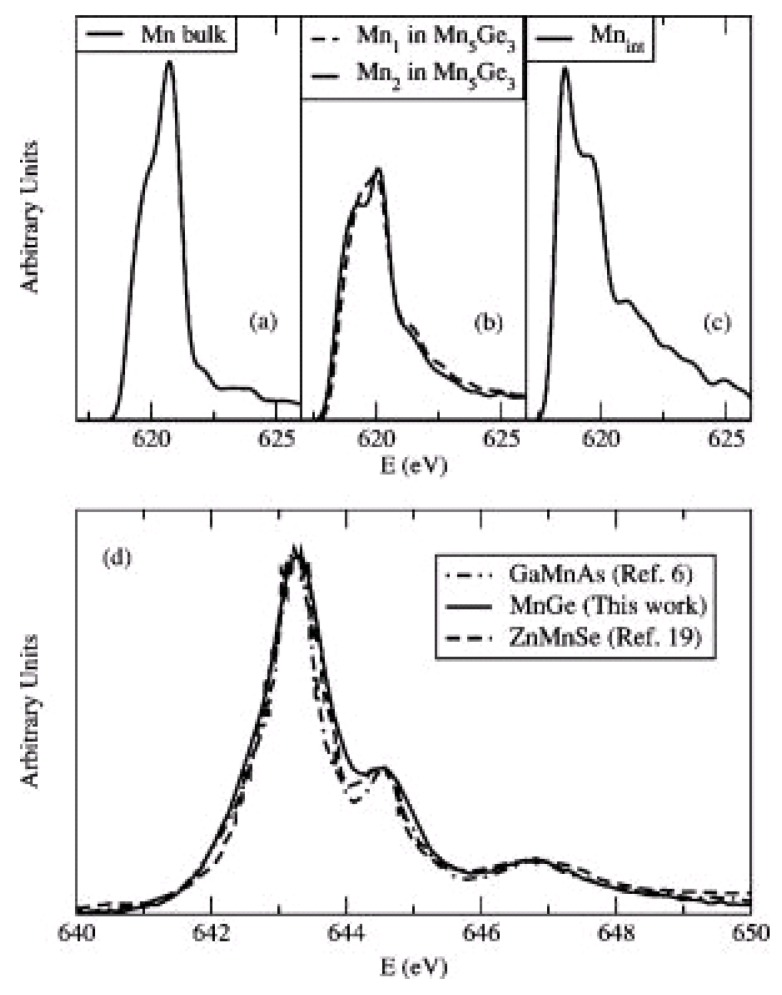
Calculated XAS spectra of (a) Mn in bulk Mn, (b) two inequivalent Mn atoms (Mn_1_ and Mn_2_) in Mn_5_Ge_3_ and (c) interstitial Mn in Mn_0.06_Ge_0.94_. (d) Experimental Mn L_3_ XAS spectrum of the Ge:Mn in comparison with Mn L_3_ XAS spectra of GaAs:Mn and ZnSe:Mn with the same Mn concentration. (Reprinted with permission from Ref. [27]. Copyright 2005, American Institute of Physics.)

As an example, in the work of Picozzi *et al*. [27] first-principle calculations of soft X-ray absorption spectra are compared with experimental data obtained for Mn-implanted Ge. The well-defined features in the spectra are recognized as a signature of homogeneous Mn dilution within the Ge host by comparing the Mn spectra in diluted MnGe alloys with other MnGe crystalline phases (see Figure 1(a–c)). On the other hand, the semiconducting host is shown to affect only slightly the Mn absorption spectrum. As shown in Figure 1(d), the Mn absorption spectra in Ge:Mn, GaAs:Mn and ZnSe:Mn are very similar to each other.

**Figure 2:**
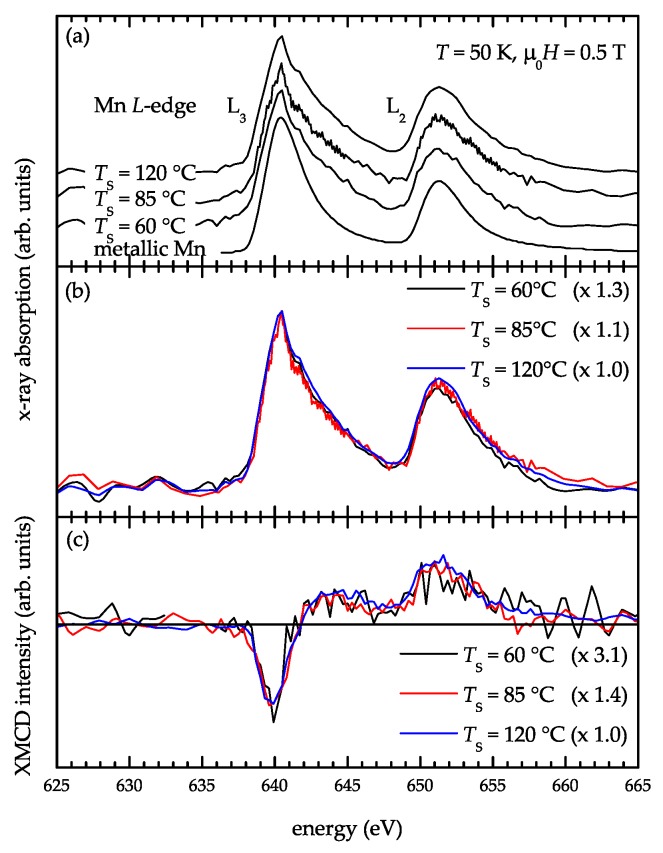
XA [(a) and (b)] and corresponding XMCD spectra (c) of GeMn thin films measured at 50 K. The total Mn content for all films is 2.8%. For comparison, the absorption spectrum of metallic Mn is included in (a). The XA and XMCD spectra are normalized to the L_3_ peak intensity of the thin film grown at 120 °C in [(b) and (c)], respectively. The scaling factors are given in the corresponding insets. (Reprinted with permission from Ref. [30]. Copyright 2009, American Institute of Physics).

However, in a recent study, Ahlers *et al.* [30] revealed an experimentally indistinguishable electronic configuration of Mn atoms incorporated in Ge_1_*_−x_*Mn*_x_* nanoclusters (a Mn diluted phase) and in precipitates of the intermetallic compound Mn_5_Ge_3_. They prepared three thin films at substrate temperatures of T*_S_* = 60, 85, and 120 °C. At T*_S_* = 60 °C, the sample consists solely of self-assembled Mn-rich Ge_1_*_−x_*Mn*_x_* nanoclusters embedded in a Ge matrix with diamond-type lattice. Increasing the fabrication temperature beyond 60 °C additionally leads to the precipitation of nanometer-sized inclusions of the intermetallic compound Mn_5_Ge_3_ in the Ge matrix. As shown in Figure 2, XAS indicates an experimentally indistinguishable charge state and local coordination of Mn in all samples. However, both XMCD as well as SQUID magnetometry measurements show that when increasing the amount of Ge_1_*_−x_*Mn*_x_* nanoclusters at the cost of Mn_5_Ge_3_ precipitates, the magnetic response of the thin films decreases.

### 2.2. Phase Separation of Mn in Ge

Although a considerable amount of Mn ions can be diluted inside the Ge matrix resulting in p-type doping, phase separation of Mn in the Ge matrix can easily happen. Depending on the preparation temperature, in most experiments Ge:Mn films contain either nanoscale Mn-rich regions or metallic precipitates such as Mn_5_Ge_3_ [31,32], Mn_11_Ge_8_ [29,33] and Mn_5_Ge_2_ [33].

**Figure 3:**
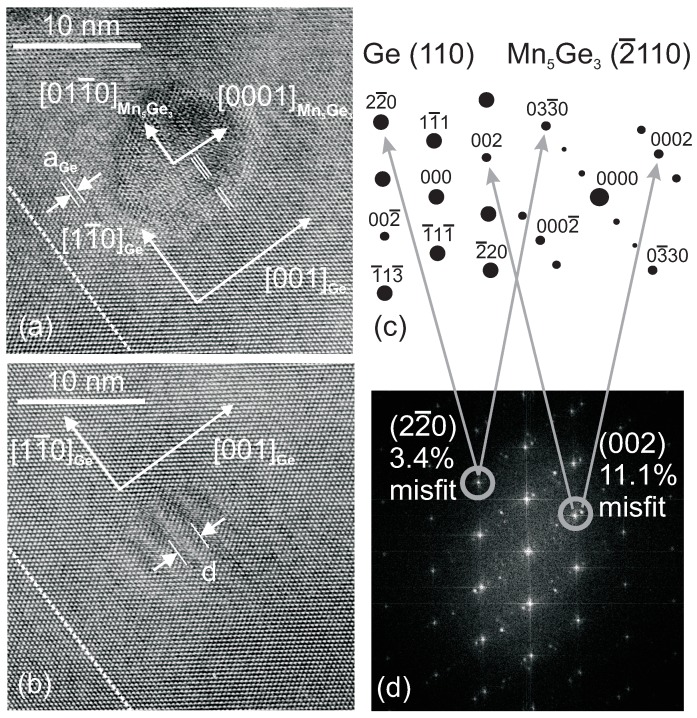
(a) and (b) HR-TEM images of typical clusters in Mn_0.03_Ge_0.97_. The orientation of the wafer-layer interface is marked by dashed lines. (c) Standard diffraction patterns of Ge(110) and Mn_5_Ge_3_(110). (d) Calculated FFT pattern of (a). (Reprinted with permission from Ref. [31]. Copyright 2006, American Institute of Physics).

In the work of Bihler *et al.* [31], a Ge:Mn film was grown by LT-MBE at 225 °C and the Mn concentration is 3%. Figure 3 shows high resolution transmission electron microscopy (HR-TEM) images of the Ge:Mn epilayer with atomic resolution. The precipitates can be identified as Mn_5_Ge_3_ with the help of the theoretical diffraction patterns shown in Figure 3(c). The Mn_5_Ge_3_ clusters were found to be preferentially oriented with their hexagonal [0001] direction aligned in the Ge[001] growth direction of the Mn_0.03_Ge_0.97_ layer.

**Figure 4:**
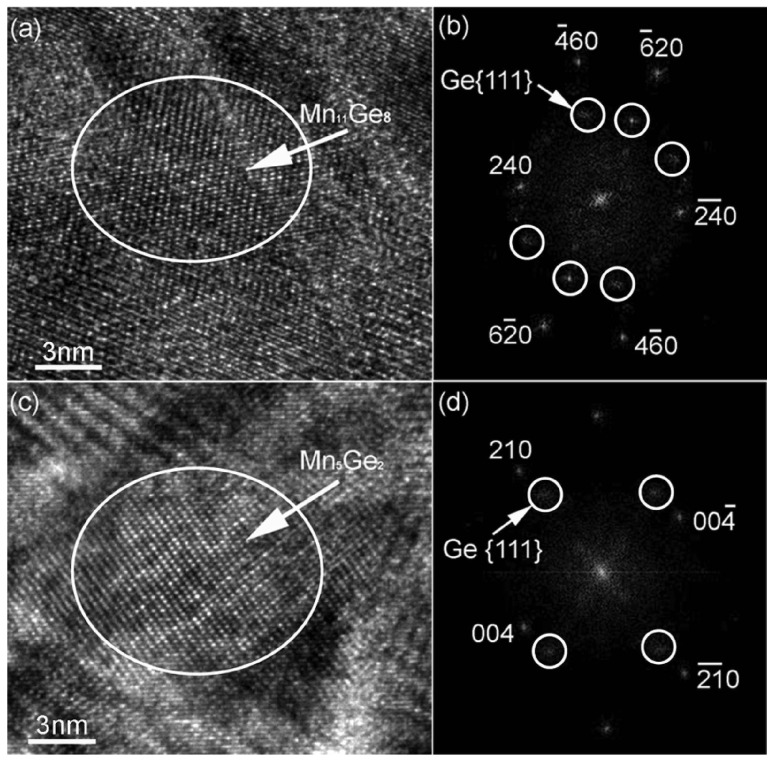
(a) HR-TEM image showing a Ge_8_Mn_11_ cluster in the thicker sample (80 nm). (b) The FFT pattern of (a). (c) Typical HR-TEM image of a Ge_2_Mn_5_ cluster in the thicker sample. (d) The FFT pattern of (c). (Reprinted with permission from Ref. [33]. Copyright 2008, American Institute of Physics).

Wang *et al.* have grown Ge_0.96_Mn_0.04_ thin films on Ge (001) substrates by a PerkinElmer solid source molecular beam epitaxy system. The growth temperature was 70 or 120 °C, although no structural difference is detectable for samples grown at different temperatures. Different from the work of Reference [31], after the growth the samples were further annealed at 400 °C for 30 min in the chamber. Two types of GeMn clusters have been observed by HR-TEM, as shown in Figure 4. They are attributed to Mn_11_Ge_8_ and Mn_5_Ge_2_.

Different from crystalline precipitates, Mn-rich Ge:Mn regions embedded inside a Mn-poor Ge matrix are often observed. The ferromagnetism in those regions is believed to be hole-mediated [21,23,34]. Nanosized Mn-rich GeMn columns in the GeMn films grown on Ge wafers result in a Curie temperature higher than 400 K.

**Figure 5:**
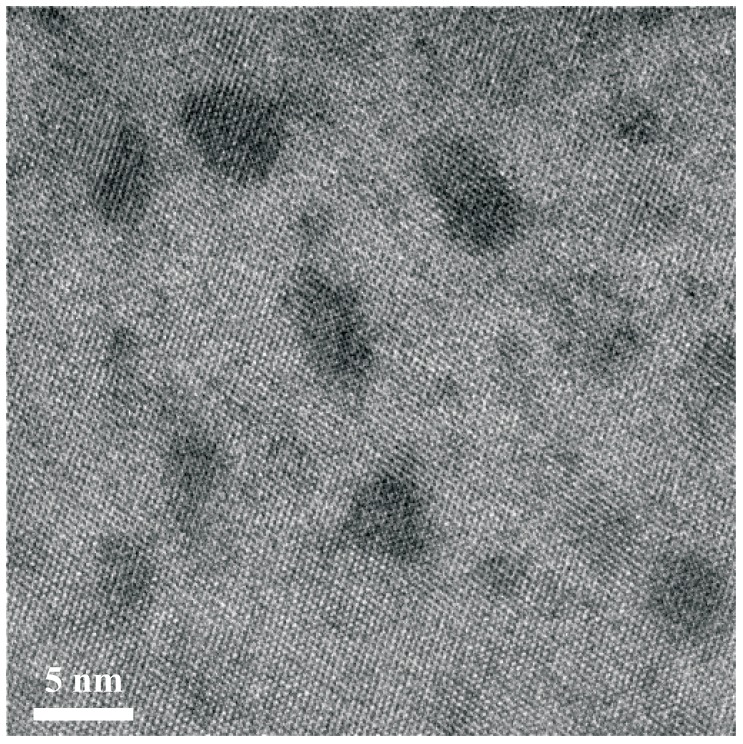
Typical HR- TEM micrograph of Ge_0.95_Mn_0.05_. Dark contrast reveals cubic clusters which are coherently bound to the surrounding matrix. (Reprinted with permission from Ref. [34]. Copyright 2006 by the American Physical Society).

Bougeard *et al.* [34] have grown Ge_0.95_Mn_0.05_ layers free of intermetallic precipitates by LT-MBE at 60 °C. As shown in Figure 5, the lattice image reveals areas with slightly darker contrast but still reflecting the same lattice symmetry. These areas are coherently bound to the surrounding Ge matrix. The upper limit of 15% Mn per cluster has been estimated.

**Figure 6:**
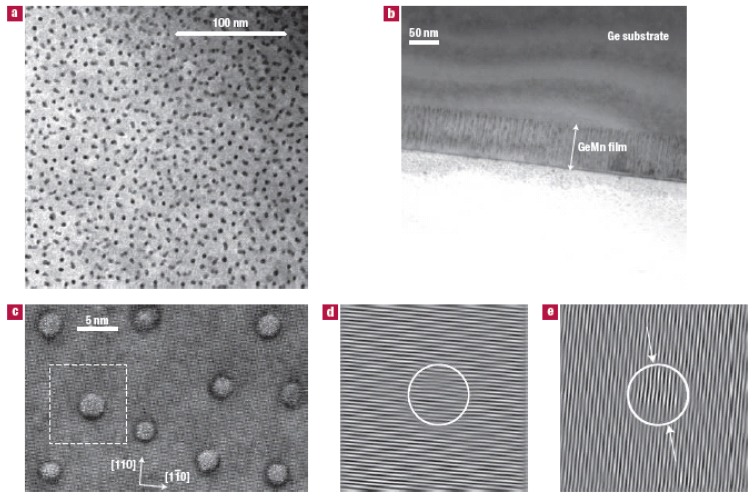
(a) Low-magnitude plane view, the dark spots are nanocolumns. (b) Low-magnitude cross-sectional image of the 80-nm-thick (Ge,Mn) layer, the nanocolumns are perpendicular to the film plane and appear as dark lines. (c) High-resolution plane view along [001] in the thinnest part of the sample. (d) and (e), Bragg filtering of (220) and (220) diffraction spots, respectively. The dashed square in (c) shows the selected area. The nanocolumn is surrounded by a white circle and the white arrows indicate the position of dislocations. A detailed explanation can be found in Ref. [23]. Reprinted by permission from Macmillan Publishers Ltd: Ref. [23].

In the work of Jamet *et al.*, (Ge,Mn) layers were grown by molecular beam epitaxy on Ge(001) substrates. They used TEM and nanoscale chemical analysis by means of electron energy-loss spectroscopy (EELS) to characterize the samples. As show in Figure 6, self-assembled nanocolumns extending through the whole thickness of the GeMn layer are clearly resolved. Mn chemical maps from EELS measurements (not shown) clearly show that the columns are Mn-rich, whereas the signal in the surrounding matrix is below the detection limit (approx 1%). Focusing on an isolated single nanocolumn in HR-TEM plane views [Figure 6(c)], around each column a dark ring reveals a large strain extending over a few interatomic distances. Fourier transform of the HR-TEM plane views reveals an isotropic distribution of the columns with a preferential spacing of 10±3 nm; their average diameter is 3±0.5 nm, and the volume fraction is 0.16. Assuming a Mn concentration between 0 and 1% in the matrix, one estimates a Mn concentration between 37.5% and 32% in the columns. The nanocolumns remain stable up to 400 °C during annealing under ultrahigh-vacuum conditions. However a 15 min annealing at 650 °C activates the volume diffusion of Mn atoms, and the nanocolumns collapse into Mn_5_Ge_3_ nanoparticles. Detailed structural and magnetic properties have been reported in References [35,36,37,38].

### 2.3. Magnetism in Ge:Mn

As shown in previous sections, in view of the crystalline structure the Ge:Mn system is highly disordered. Three kinds of phases can form depending on preparation and annealing temperature: the Mn diluted Ge in which the Mn concentration seems well below 1%, Mn-rich Ge:Mn nanostructures and MnGe crystalline precipitates. Consequently, the magnetic properties of Ge:Mn systems also reveal a multi-phase nature. Of course, it might be difficult to distinguish between the Mn diluted Ge and Mn-rich Ge:Mn nanostructures.

**Figure 7:**
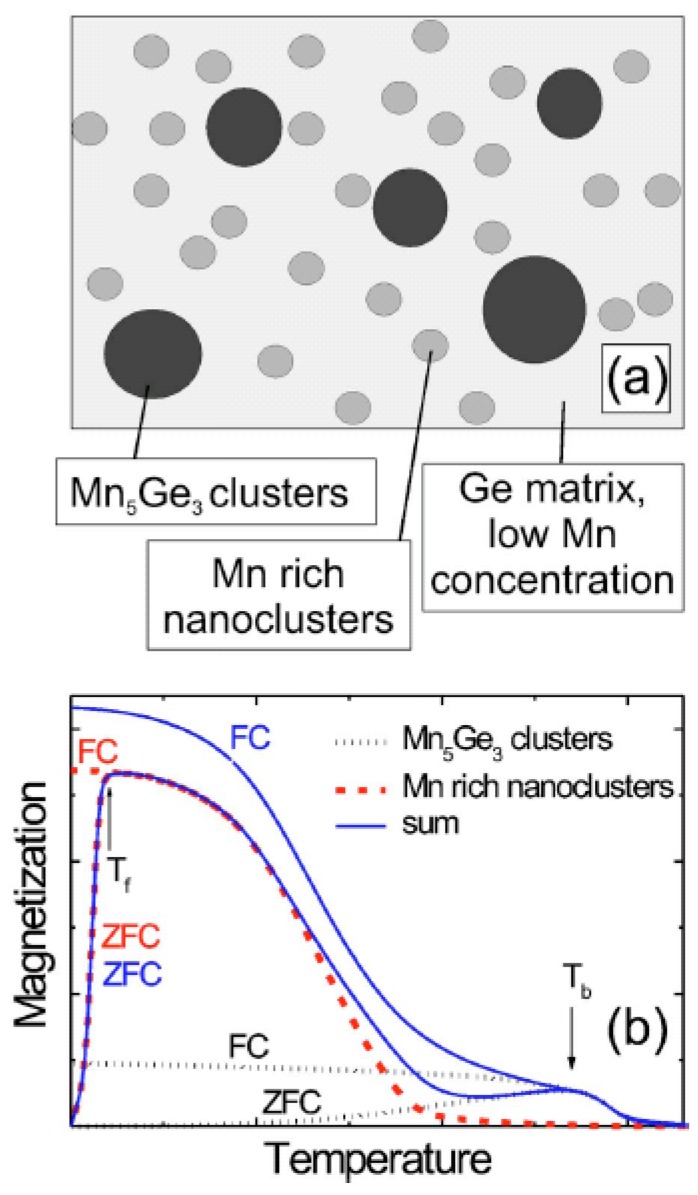
(a) Illustration of the two different kinds of clusters present in Ge:Mn samples. (b) Schematic FC (field cooling) and ZFC (zero field cooling) magnetization curves for both kinds of clusters: black dotted curves for the contribution of the Mn_5_Ge_3_ clusters, red dashed curves for the Mn-rich nanoparticles and solid blue curves for the sum. (Reprinted with permission from Ref. [39]. Copyright 2006 by the American Physical Society).

In the work of Jaeger *et al.*, a nice illustration of structural and magnetic phases has been given (see Figure 7). Two transition temperatures T*_f_* and T*_b_* are attributed to blocking or freezing transitions of two different kinds of superparamagnetic Mn-rich nanoclusters and Mn_5_Ge_3_ clusters, respectively. In Figure 7(b) the expected (Field cooled) FC and zero-field cooled (ZFC) magnetization curves for the Mn-rich nanoclusters (red dashed curves) and the Mn_5_Ge_3_ clusters (black dotted curves) are plotted schematically. The sum of both FC and ZFC contributions (blue solid curves) qualitatively explain the experimentally observed FC and ZFC measurements. However, in Figure 7 the Mn-diluted Ge is not specified.

**Figure 8:**
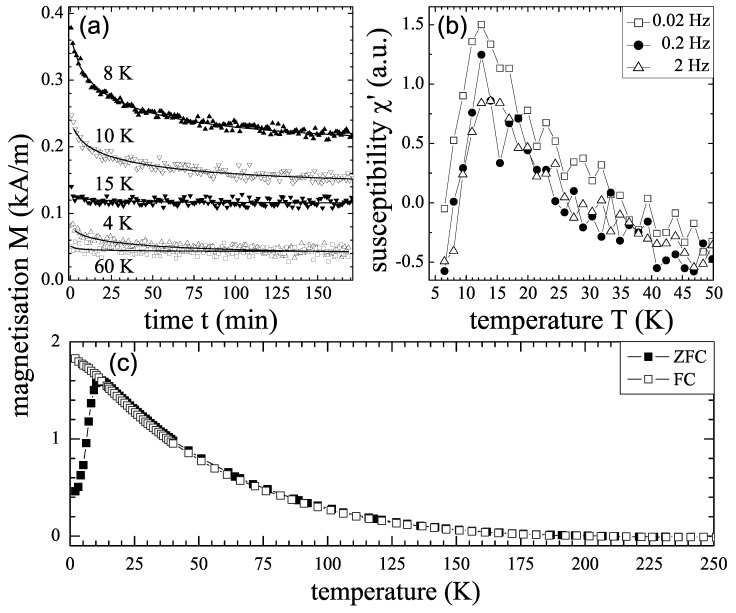
(a) Relaxation of the magnetization of Ge_0.95_Mn_0.05_ after switching off the external field at different temperatures. Solid lines represent fits to a stretched exponential decay. (b) Temperature-dependent real part of the ac susceptibility measured at different frequencies. (c) FC or ZFC temperature-dependent magnetization in a field of 0.1 T. (Reprinted with permission from Ref. [34]. Copyright 2006 by the American Physical Society).

**Figure 9:**
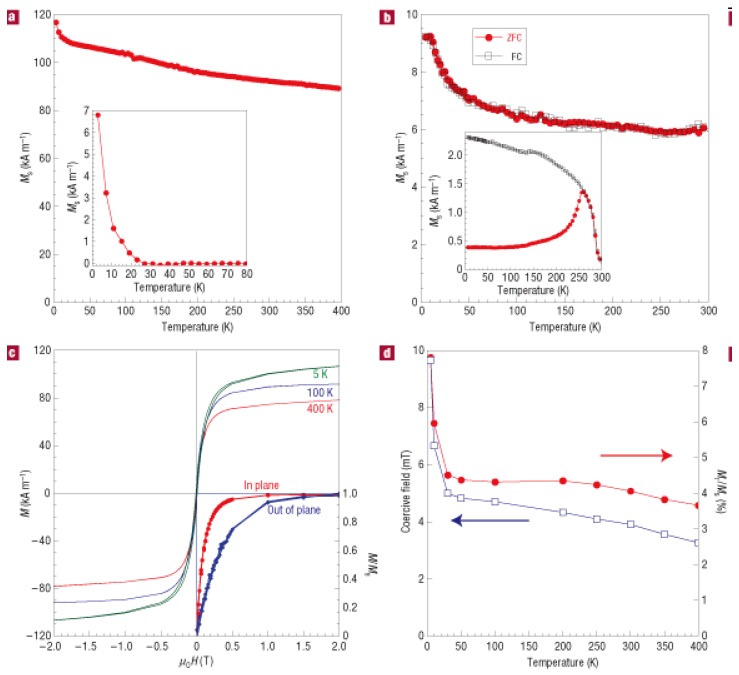
(a) Temperature dependence of the saturation magnetization measured at 2 T. The inset shows the extrapolated matrix signal at low temperature after subtracting the nanocolumns magnetic signal. (b) ZFC-FC measurements carried out at 0.01 T. Both curves superimpose. Inset: ZFC-FC curves after 15-mins annealing at 650 °C. (c) Magnetization loops at 5, 100 and 400 K, after subtracting the diamagnetic contribution from the substrate. The inset demonstrates the easier saturation in-plane at 250 K. (d) Coercive field and remanent magnetization versus temperature. Reprinted by permission from Macmillan Publishers Ltd: Ref. [23], copyright 2006.

We first discuss the magnetic properties of Mn diluted Ge or Mn-rich Ge:Mn nanostructure. Often, a low-temperature (10–20 K) magnetic phase is observed in Ge:Mn samples prepared by MBE or by ion implantation [23,34,40,41,42]. As an example, Figure 8 shows the magnetic properties of a Ge_0.95_Mn_0.05_ film. Its structural properties have been shown in Figure 5. The films show no overall spontaneous magnetization at all down to 2 K. The TEM and magnetization results can be understood in terms of an assembly of superparamagnetic moments developing in the dense distribution of clusters. Each cluster individually turns ferromagnetic below an ordering temperature which depends on its volume and Mn content. Figure 9 shows the magnetic properties of the Ge:Mn film consisting of Mn-rich Ge:Mn nanocolumns (see Figure 6). The inset of Figure 9(a) shows the magnetic phase with a lower ordering temperature, which is attributed to the Mn-poor Ge:Mn matrix. If we believe that the Mn ions in Mn-rich Ge:Mn nanostructures are also substitutional, one might get the following thumb rule after considering the magnetic properties presented in References. [21,23,34]. The ordering temperature is increased with increasing the diluted Mn concentration. To realize a ferromagnetic phase above room temperature, one needs a Mn concentration above 30%, which of course depends on the size of Mn-rich nanostructures.

Secondly, we discuss the magnetic properties of the system consisting of MnGe precipitates. An ensemble of nanomagnets exhibits rich magnetic properties, with large technological impact. Temperature-dependent memory effects and slow magnetic relaxation have been observed in a GaAs:Mn system containing Mn-rich clusters [43]. Such kind of effect has been observed also in Mn_5_Ge_3_ nanomagnets embedded inside a Mn-diluted Ge matrix [42].

**Figure 10:**
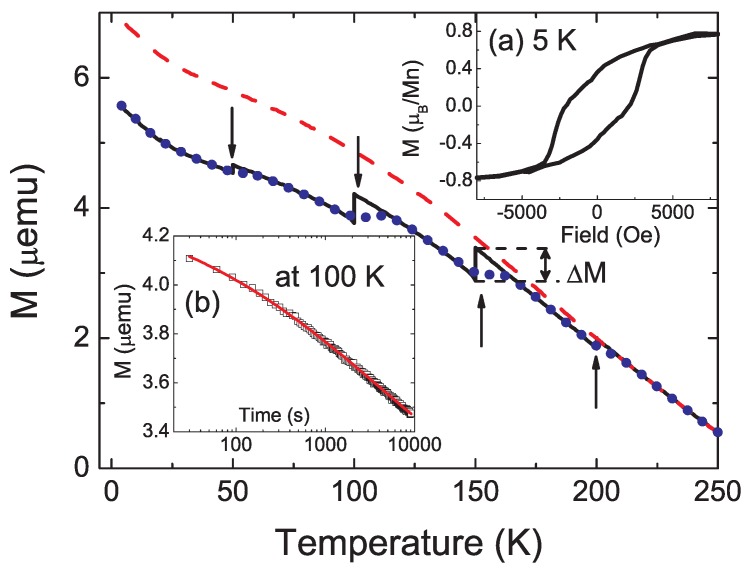
Temperature dependent memory effect in the dc magnetization of the GeMn precipitates inside the Ge matrix. The dashed line (red) is measured during cooling in 50 Oe at a cooling rate of 1 K per minute, while the solid line (black) is measured in 50 Oe with the same cooling rate but with a stop of 2 hours at 200 K, 150 K, 100 K, 50 K and 20 K. The field is cut off during stop. The dotted line (blue) is measured with continuous heating at the same rate after the previous cooling protocol. Inset (a): Hysteresis loop measured at 5 K. Inset (b): Time dependent remanent magnetization measurement after cooling from 300 K to 100 K with a field of 50 Oe. Scattered symbols are experimental data and the solid line (red) is a fitting using the stretched-exponential function. (Reprinted with permission from Ref. [42]. Copyright 2009, American Institute of Physics).

As shown in Figure 10, the history-dependent magnetic memory measurements were performed using a cooling and waiting protocol suggested by Sun *et al.* [44]. We cooled the sample at 50 Oe and recorded the magnetization with cooling, but temporarily stopped at 200 K, 150 K, 100 K, 50 K and 20 K for a waiting period of 2 hours. During waiting, the field was set to zero. After the stop, the 50 Oe field was re-applied and cooling and measuring were resumed. The temporary stops resulted in a steplike M(T) curve (solid line, black) in Figure 10. After reaching 4 K, the sample was heated back in the same field, and the magnetization was recorded again (dotted line, blue). During this heating the M(T) curve also has a steplike behavior at the stop temperatures, then recovers the previous M(T) curve measured during cooling, *i.e.* the system remembers its thermal history. The steplike feature in the temperature dependent magnetization is a result of magnetic relaxation at the stopping points [44]. This observation clearly demonstrates that nanomagnets embedded inside the Ge matrix behave like a spin-glass.

### 2.4. Magnetotransport

A diluted ferromagnetic semiconductor should exhibit strong magneto-transport effects, namely negative magnetoresistance (MR) and anomalous Hall effect (AHE) [45,46,47], and provide the possibility to control the spin by an external electric field. For ferromagnetic GaMnAs, usually the AHE is taken as a measure of its magnetization. The observation of AHE is considered as one of the important criteria for FMS to be intrinsic [48].

We notice that pronounced MR and AHE have been reported in the Ge:Mn system [20,22,23,49,50,51,52,53] independent of the formation of MnGe precipitates or not, as well as in Cr doped Ge [54]. By scrutinizing the published data on Ge:Mn, one can observe three features in the reported AHE. First, most of the AHE curves shown were recorded at temperatures above 10 K. Indeed, Riss *et al.* [50] reveal only ordinary Hall effect below 10 K. Second, no hysteresis in AHE curves has been observed, despite the observation of a clear hysteresis in magnetization, which is much different from the case of III-Mn-V and ZnMnTe [45,46,47]. Third, the Hall curve changes its sign of slope at lower temperatures, usually between 10 K and 50 K. Obviously, the correlation between magnetization, MR, and AHE, which is a hallmark of III-Mn-V and ZnMnTe FMS [45,46,47], has not been proven for Ge:Mn so far. As we showed in Section 2.2., Mn-phase segregation is difficult to avoid. Even at a growth temperature as low as 70 °C, Mn-rich nanostructures can form. This arises a question: if the Mn-rich nanostructures are not percolating, one only measures the transport properties of the Mn-poor matrix. This fact may explain the lack of hysteretic magneto-transport properties in the Ge:Mn system. In the work of Zhou *et al.*, the two-band-like conduction in semiconductors has been considered to explain the observation of anomalous Hall resistance in the Ge:Mn system.

**Figure 11:**
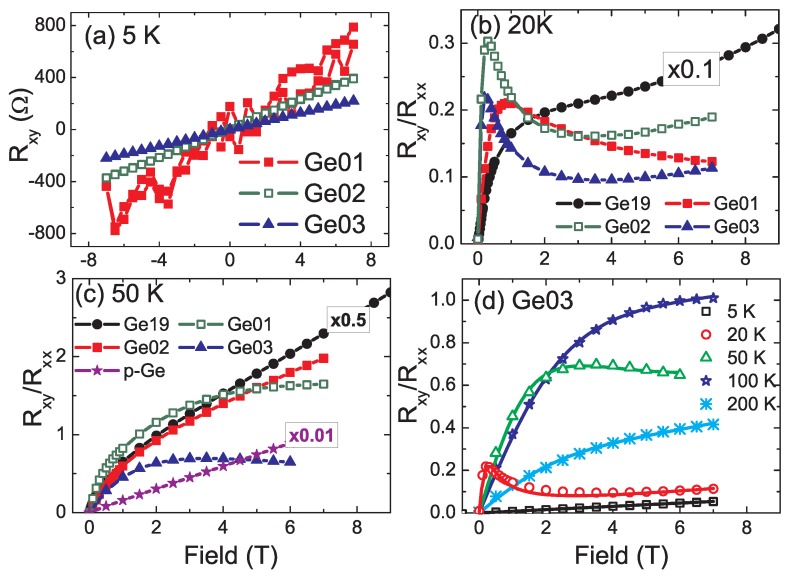
(a) Hall resistance (*R_xy_*) at 5 K of the Ge:Mn samples listed in Table 1: only ordinary Hall effect has been observed. (b) and (c): The ratio between *R_xy_* and sheet resistance at zero field (*R_xx_*) at 20 K and 50 K, respectively. An anomalous Hall resistance appears and the sign of slope is changed at lower fields (20 K) or at larger fields (50 K). (d) *R_xy_*/*R_xx_* at different temperatures for sample Ge03: the symbols are experimental data, while the solid lines are fits using a two-band-conduction theory. A Ga-doped Ge wafer [p-Ge in (c)] with a hole concentration of 1.5×10^16^ cm^−3^ was measured for comparison and only ordinary Hall effect is observed as shown in (c). For better visibility some curves are multiplied by the factors indicated. (Reprinted with permission from Ref. [55]. Copyright 2009, American Institute of Physics).

In the work of Zhou *et al.*, intrinsic Ge(001) wafers were implanted with Mn ions at 300 °C to avoid amorphization. The ion fluence was varied to get samples with a large range of Mn concentrations and correspondingly different structural and magnetic properties (see Table 1). Down to 5 K, only diamagnetism was probed for sample Ge01, identical to a virgin Ge sample. Note that independent of the formation of precipitates, a fraction of Mn ions has been confirmed by spectroscopic methods to be diluted inside the Ge matrix, resulting in p-type doping [27,28,29], as well as by electrical transport measurements as shown in Figure 11(a). In sharp contrast to the structural and magnetic properties, similar Hall effects are probed for all samples as shown in Figure 11. The non-ferromagnetic nature of sample Ge19 and Ge01 indicates that the observation of anomalous Hall resistance is not necessarily related to ferromagnetism. Actually, similar Hall curves have been observed in materials with a two-band-like conduction [56,57,58]. As shown in Figure 11(d), the Hall curve can be well fitted by considering two types of carriers with different mobility and population.

**Table 1:** Sample identification (ID), Mn concentration (Mn conc.), sheet hole concentration (Hole conc.) and magnetic properties. (Reprinted with permission from Ref. [55]. Copyright 2009, American Institute of Physics.)

ID	Mn conc.	Hole conc.	Properties	
	%	cm^−2^	Precipitates	Ferromagnetic
Ge19	0.004	-	No	No
Ge01	0.2	6.5×10^12^	No	No
Ge02	2	1.1×10^13^	Mn_5_Ge_3_	Yes
Ge03	10	2.0×10^13^	Mn_5_Ge_3_	Yes

### 2.5. Pulsed Laser Annealing of Mn Implanted Ge

Until now, we are focusing on samples prepared either by LT-MBE or by ion implantation at elevated temperatures. By ion implantation at low temperature (for instance by cooling the substrate with liquid N_2_ flow), one can introduce enough Mn ions inside the Ge matrix and simultaneously avoid secondary phase formation. However, the goal is not only to introduce a large number of dopant atoms, but also to ensure that these atoms are electrically active. Therefore, one needs an effective annealing method, which can recrystallize the implanted materials, activate the dopants, and at the same time, suppress the formation of secondary phases. Pulsed laser annealing (PLA) provides such a possibility. Shallow dopants in Si and Ge, mainly the elements from the III or V columns of the periodic table, have been successfully built in by ion implantation and PLA. The carrier concentration can reach values as high as 10^21^ cm^−3^ [59]. The success stands on the fact that the large energy deposition within nano-seconds from a pulsed laser creates a liquid Si or Ge in the implanted, near surface region, and the large cooling rate (10^11−13^ K/s) suppresses diffusion and precipitations.

In our preliminary experiments [60] the hole concentration in Mn-implanted Ge can be strongly increased by PLA. The largest hole concentration achieved is around 2.1 × 10^20^ cm^−3^ in samples after pulsed laser annealing (PLA), showing negative MR and AHE with the same hysteresis as the magnetization at low temperatures.

In this experiments, nearly intrinsic, n-type Ge(001) wafers were implanted with Mn ions. The implantation energy and fluence were 100 keV, 30 keV and 5×10^16^ cm^−2^, 1×10^16^ cm^−2^, respectively, resulting in a box-like distribution of Mn ions with concentration around 10 % over a depth of 100 nm. During implantation the wafers were flow-cooled with liquid nitrogen to avoid the formation of any Mn-rich secondary phase. Pulsed laser annealing was performed using a laser ASAMA 80-8 and an optical system VOLCANO from INNOVAVENT GmbH. The pulse duration was 300 ns at a wavelength of 515 nm. The introduced energy density was 1.5 J/cm^2^.

**Figure 12:**
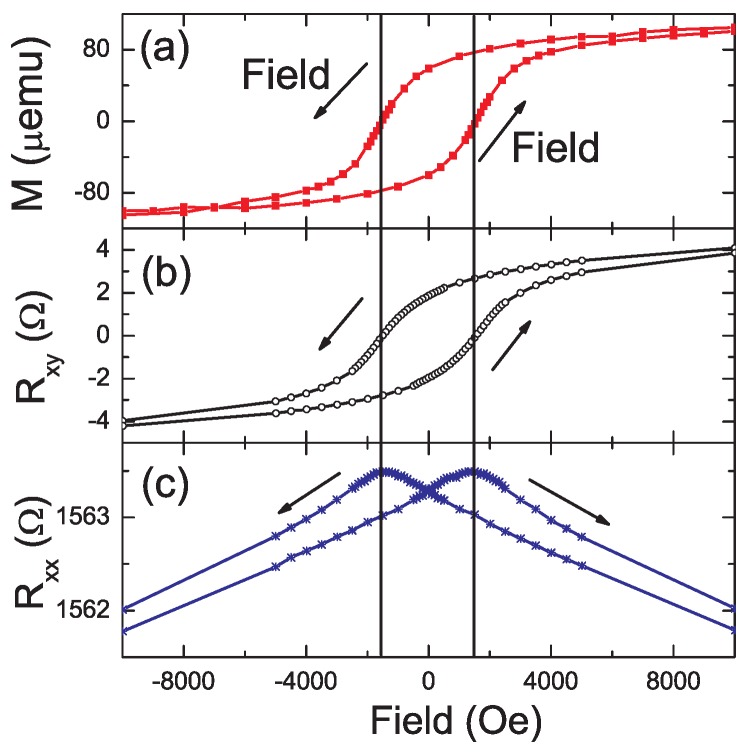
The field dependent (a) magnetization (*M*), (b) Hall resistance (*R_xy_*) and (c) longitudinal resistance (*R_xx_*) of the Ge:Mn system at 5 K. Hysteresis appears for both *R_xx_* and *R_xy_* curves, with the same coercivity as for *M*. The longitudinal resistivity is around 0.015 Ω-cm if assuming a thickness of 100 nm. (Reprinted with permission from Ref. [60]. Copyright 2010 by the American Physical Society).

Figure 12 shows the comparison of the field-dependent magnetization, Hall resistance, and longitudinal resistance at 5 K measured on the Ge:Mn sample after PLA. The involvement of holes in the ferromagnetism is unambiguously confirmed by the appearance of the AHE, and, especially, by the clear hysteresis loop in the Hall resistance. Note that the presence of ferromagnetic characteristics in SQUID measurements alone could also be caused by ferromagnetic precipitates, e.g., Mn_5_Ge_3_, in addition to or instead of a FMS. At low temperature, hysteresis appears in both the Hall and longitudinal resistance curves, with the same coercive field as in the magnetization. Such a correlation between AHE, MR, and magnetization is usually considered as the signature of FMS, [46,47] where the same set of holes contribute to ferromagnetism and transport [10]. The Curie temperature is around 7.5 K. This also has been observed in compensated GaAs:Mn [61] and insulating GaP:Mn [62]. However, to our knowledge, the correlation between AHE, MR, and magnetization has never been observed for other Ge:Mn samples. The hole concentration plays a critical role in establishing carrier-mediated ferromagnetism in magnetic semiconductors [63].

## 3. Mn-Doped Si

To dope Mn in Si is even more challenging. The behavior of transition metals in Si under equilibrium conditions has been well documented in the 1980s. Several conclusions have been drawn in the paper by Weber [64]:

- 3d transition metals diffuse predominantly as interstitials in Si with an activation energy of diffusion near 0.75 eV, and stay in interstitial sites at high temperature in thermal equilibrium;
- V, Cr, Mn, and Fe can be quenched in interstitial sites of tetrahedral symmetry, and Co, Ni, and Cu vanish out of the interstitial solutions during quenching;
- Energy levels for the interstitial species and various pairs have been established, based mainly on combinations of different experimental methods with EPR results;
- Substitutional 3d metals in silicon are rather exotic species, produced under non-equilibrium conditions like irradiation. Their energy levels have not been established.

Using density-functional theory, Wu *et al.* [65] demonstrated that interstitial Mn can be utilized to tune the magnetic properties of Si. Experimentally, various groups have reported the observation of ferromagnetism in Mn-doped Si [66,67,68,69,70,71]. The reported Curie temperatures range from 200 to 400 K. However, the opinions concerning the origin of the observed ferromagnetism are very diverse. In the following sections we discuss the structural and magnetic properties of Mn-doped Si.

### 3.1. Mn in Si: Substitutional?

Compared with Mn in Ge, the knowledge about the lattice location and electronic structure of Mn in the Si matrix is more contradictive. In last years, two papers completely contradicting with each other, have been published.

**Figure 13:**
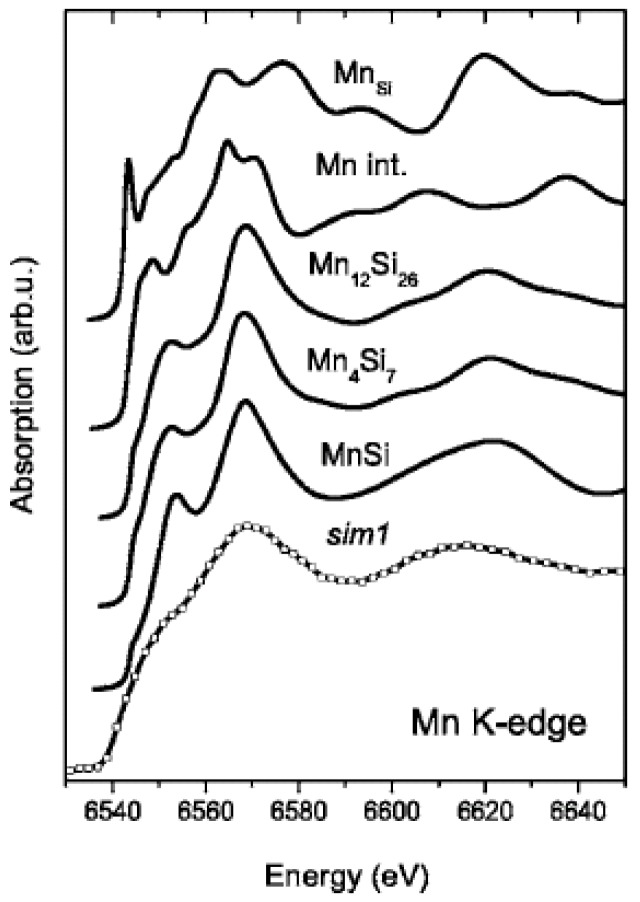
Experimental spectrum for Mn implanted Si and XANES spectra calculated for different models of Mn location in the Si lattice: Mn*_Si_*, substitutional; Mn*_int_*, interstitial; Mn_12_Si_26_, Mn_4_Si_7_, and MnSi, the order as in crystalline Mn-Si compounds. The implantation fluence was 1 × 10^16^*cm*^−2^ and the implantation temperature was 340 K. (Reprinted with permission from Ref. [72]. Copyright 2008 by the American Physical Society).

In the work of Wolska *et al.* [72], the Si:Mn samples were prepared by Mn-ion implantation into Si. The implantation temperatures were 340 K or 610 K. They investigated the local order around Mn atoms in the ferromagnetic Mn-implanted Si samples using of X-ray absorption spectroscopy techniques (see Figure 13). Analysis of both extended X-ray absorption fine structure and X-ray absorption near-edge structure spectra clearly indicates that Mn ions are located neither in the substitutional nor in the interstitial position in the Si lattice. Depending on how the samples were prepared, they have five to eight nearest neighbors.

**Figure 14:**
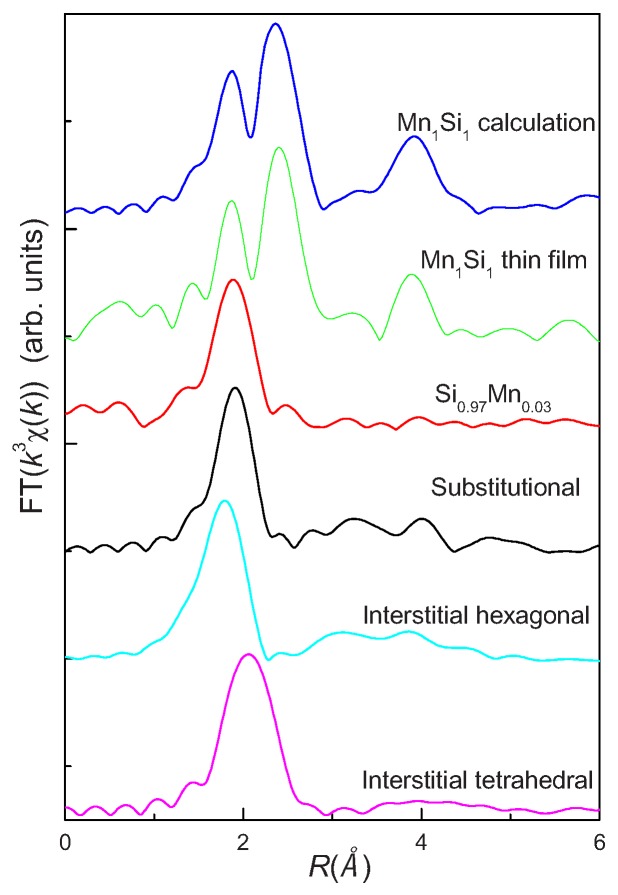
Comparison of the radial structural functions from theoretical calculations and the experimental radial structural functions of the Si_1_*_−x_*Mn*_x_* thin films. It can be clearly found that the experimental spectrum with a strong peak (1.90 *Å*) in the Si_1_*_−x_*Mn*_x_* thin film is exactly reproduced by the calculated spectrum for substitutional MnSi. (Reprinted with permission from Ref. [73]. Copyright 2009, American Institute of Physics).

However, in the work of Ye *et al.* [73], Mn-doped Si thin films were prepared by a magnetron cosputtering method at a low growth temperature. They concluded that the doped Mn ions substitute for Si sites based on a detailed analysis of the extended X-ray absorption fine structure (XAFS) together with the X-ray absorption near-edge structure spectra at the Mn*_K_*-edge (see Figure 14).

### 3.2. Mn-Silicide Formation

In two pioneering works concerning Mn-doped Si prepared by ion implantation [67] or sputtering [66], ferromagnetism was reported, however, a detailed structural characterization was lacking. Later on, using high resolution, spatially resolved techniques, comprehensive material characterization reveals the clustering of Mn-rich phases in Mn implanted Si, namely MnSi_1.7_ [68,69,71,74,75], which is the energetically most favorable Mn-silicide phase [76,77].

The work of Ko *et al.* represents a detailed TEM characterization on Mn implanted Si. They employed several advanced techniques including electron energy loss spectroscopy, Z-contrast scanning TEM imaging, and electron diffraction to scrutinize the secondary phase formation in Mn implanted Si.

**Figure 15:**
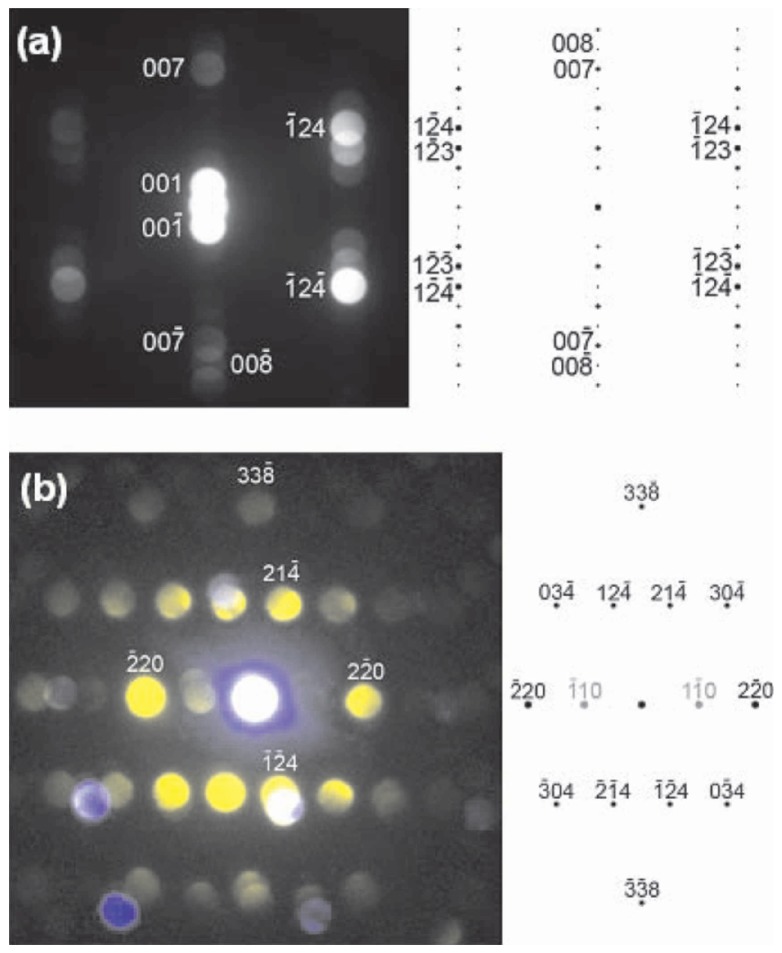
(a) [210] CBED pattern from a Mn_4_Si_7_ precipitate in Mn implanted Si and the corresponding simulation, and (b) [443] CBED pattern from a Mn_4_Si_7_ precipitate and the corresponding simulation (in the experimental pattern spots from the Si matrix appear blue and those from the precipitate are yellow). (Reprinted with permission from Ref. [69]. Copyright 2008, American Institute of Physics).

**Figure 16:**
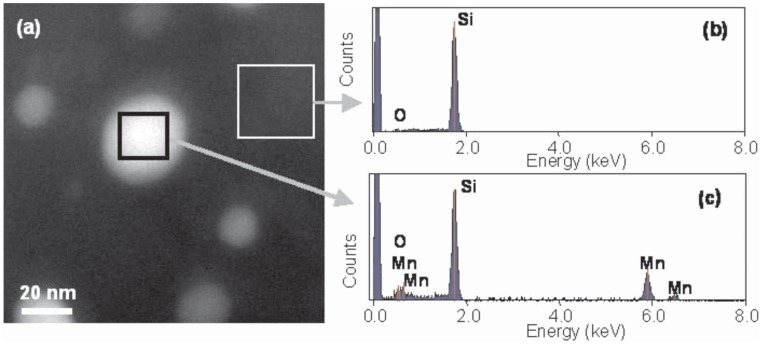
(a) HAADF STEM image of precipitates in Mn implanted Si. [(b) and (c)] The EDX spectra obtained by scanning the beam in the regions indicated by the boxes on (a). (Reprinted with permission from Ref. [69]. Copyright 2008, American Institute of Physics).

Figure 15 shows the convergent beam electron diffraction (CBED), revealing the crystallographic structure of randomly chosen precipitates. Figure 15(a) shows a [210] diffraction pattern clearly exhibiting periodicities related to the long c-axis of the Mn_4_Si_7_ structure, accompanied by a simulation. Figure 15(b) shows a [443] diffraction pattern colored in yellow, which is also characteristic of the Mn_4_Si_7_ structure. The diffraction patterns of several other precipitates were indexed and also showed good matches with Mn_4_Si_7_ as did selected area diffraction patterns. Fast Fourier transforms of HR-TEM images from some of the precipitates are also indexed as Mn_4_Si_7_. They concluded that many of the precipitates are of the tetragonal Mn_4_Si_7_ phase. Using chemical composition of analysis, the precipitates were confirmed to be Mn-rich (see Figure 16).

**Figure 17:**
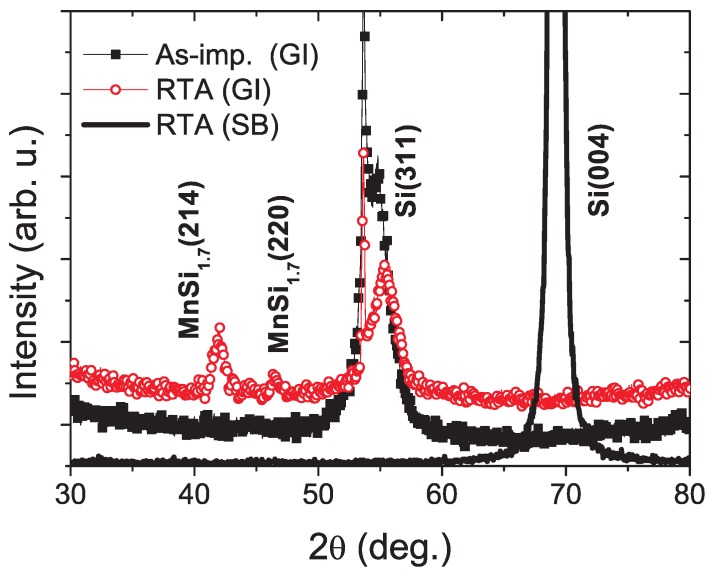
XRD patterns of Mn implanted Si. GI: grazing incidence, and SB: symmetric beam path. The diffraction peaks at around 42° and 46.3° cannot be attributed to the Si substrate, but to MnSi_1.7_. (Reprinted with permission from Ref. [68]. Copyright 2007 by the American Physical Society).

The work of Zhou *et al.* represents an example of structural characterization of Mn implanted Si using X-ray diffraction [68]. They show that a lab-equipped X-ray source may be not sensitive enough to detect tiny nanocrystalline particles. Using synchrotron radiation X-ray diffraction in grazing incidence geometry, MnSi_1.7_ silicide nanoparticles are detected as shown in Figure 17.

### 3.3. Magnetic Properties

Corresponding to the observation of phase separation of Mn in Si, the interpretation of the magnetic properties also becomes complex.

**Figure 18:**
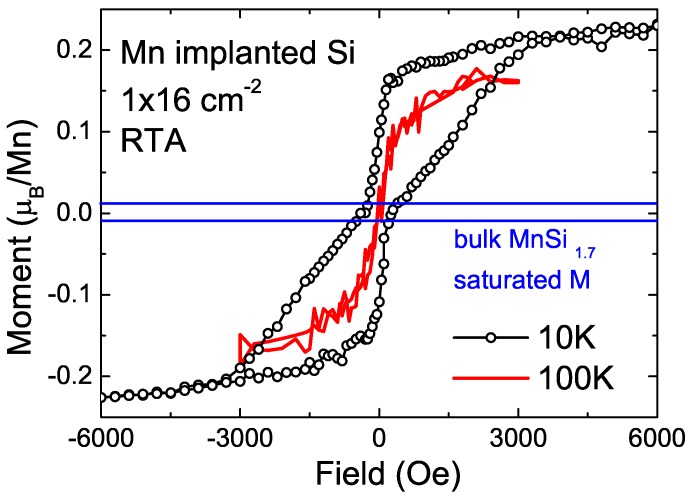
Magnetic properties of Mn implanted Si measured at 10 and 100 K. The saturation magnetization of MnSi_1.7_ is indicated by lines for comparison. (Reprinted with permission from Ref. [68]. Copyright 2007 by the American Physical Society).

**Figure 19:**
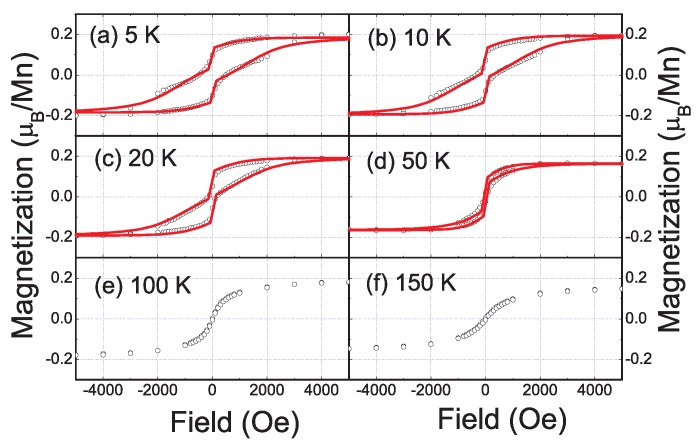
Hysteresis loops (open circles) measured in the temperature range from 5 K to 150 K. The solid curves (a–d) are fittings using the Preisach model. (Reprinted with permission from Ref. [80]. Copyright 2009 by the American Physical Society).

Zhou *et al.* and Yabuuchi *et al.* attributed the observed ferromagnetism to MnSi_1.7_ nanoparticles. If assuming all Mn ions form MnSi_1.7_ nanoparticles, they got a saturated magnetization of 0.21 *µ_B_*/Mn. It is much larger than that in bulk MnSi_1.7_, as shown in Figure 18. Of course, one can question if there is another contribution to the observed ferromagnetism. However, the wasp-waist shape of the loop is associated with magnetic phases with different coercivities. The size distribution of MnSi_1.7_ nanoparticles can result in a distribution of coercivities. Using the Preisach model [78,79], the magnetic properties at different temperature can be well explained by solely considering the contribution of MnSi_1.7_ nanoparticles (see Figure 19). Using first-principles calculation, Yabuuchi *et al.* have clarified that the stoichiometry, strain and charge accumulation as well as the interface between MnSi_1.7_ and Si strongly influence the magnetic properties of MnSi_1.7_ nanoparticles [70]. These effects well account for the experimental observations.

**Figure 20:**
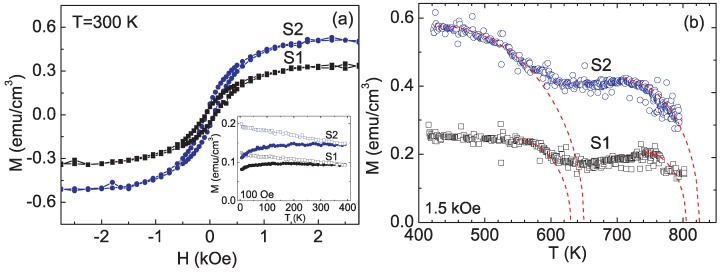
(a) M–H curves at 300 K for Mn implanted Si (S1: 0.4% Mn, and S2: 1.8% Mn). The inset shows their ZFC (open circles)-FC (filled circles) curves from 5 to 390 K under an applied field of 100 Oe. (b) M-T curves from 410 to 800 K for S1 and S2 samples under an applied field of 1.5 kOe. The red dashed lines are fits to M*_s_*(T) = M_0_[1-(T/T*_C_*)2]1/2. (Reprinted with permission from Ref. [69]. Copyright 2008, American Institute of Physics).

At the end, we would not completely exclude other contributions to the ferromagnetism observed in Mn-doped Si. In the work of Ko *et al.*, the temperature dependent magnetization indicates multifold contributions. As show in Figure 20(a), the hysteresis (M-H) loops measured at 300 K before the Mn implanted Si were annealed revealed the ferromagnetic ordering around room temperature. After annealing, the magnetization increased by about a factor of 3. The low value of magnetization in the samples was interpreted to the presence of antiferromagnetic coupling between the Mn moments and an appreciable number of magnetically inactive Mn atoms. The ZFC-FC curves measured at 100 Oe for S1 and S2 samples (S1: 0.4% Mn, S2: 1.8% Mn) are shown in the inset of Figure 20(a). One can clearly see that the ferromagnetic ordering persists up to 390 K. A divergence between the ZFC and FC curves at low temperatures as well as reversibility above 350 K are observed. Figure 20(b) shows the temperature-dependent magnetization curves measured from 410 to 800 K. The magnetizations are found to persist up to even 800 K for both S1 and S2 samples. The magnetization curves show a significant change in slope near 600 and 750 K. Two transition temperatures are obtained to be around 630–650 K and 800–830 K, respectively, by fitting with the Brillouin function.

### 3.4. Magnetotransport Properties

Comparing with the magnetic properties of Mn-doped Si, the magnetotransport properties are rarely investigated. This is also a hint that it is still far away to realize Si-based ferromagnetic semiconductors.

**Figure 21:**
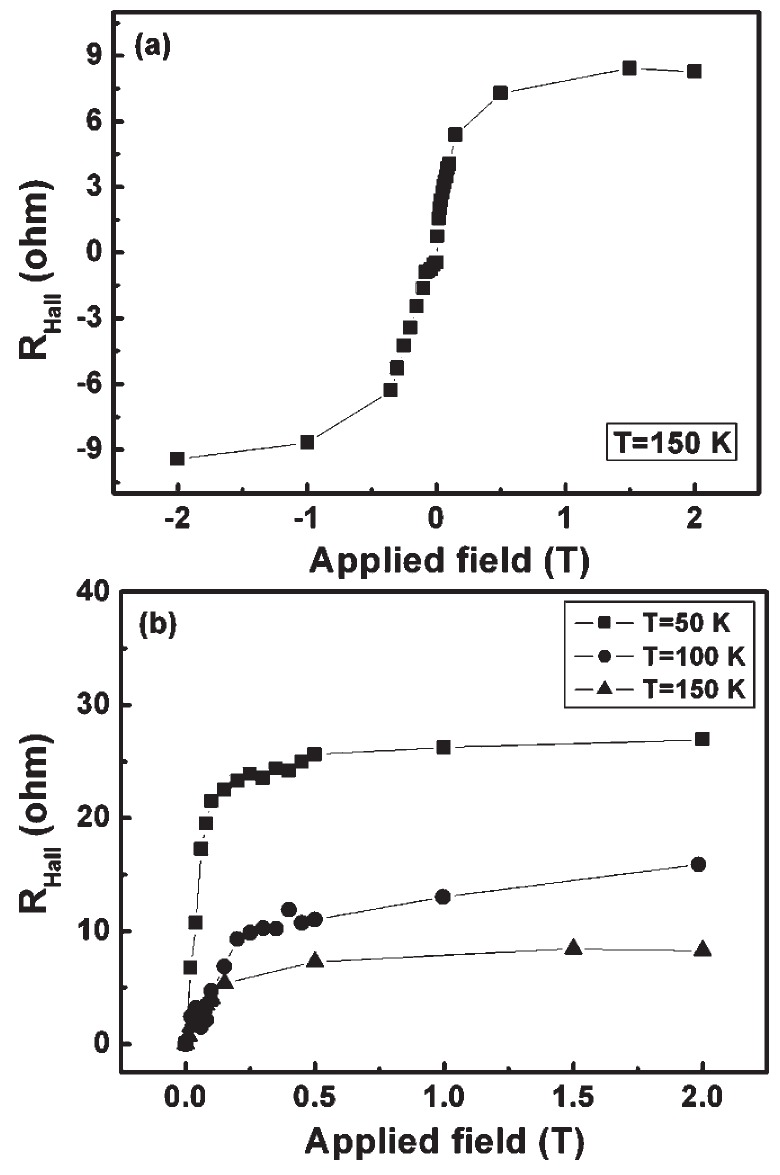
The whole Hall resistance *vs*. field of an a-Si_1_*_−x_*Mn*_x_* sample measured at 150 K. (b) Collective Hall resistance: Hall vs field curves of an a-Si_1_*_−x_*Mn*_x_* (x = 0.105) sample measured at 50, 100, and 150 K, respectively. (Reprinted with permission from Ref. [81]. Copyright 2009, American Institute of Physics).

Yao *et al.* prepared amorphous Si_1_*_−x_*Mn*_x_* thin films with hydrogen on SiO_2_/Si (100) substrates at room temperature by the magnetron cosputtering method [81]. The films are very conductive with carrier concentrations up to 10^20^ cm^−3^. As shown in Figure 21, anomalous Hall effect is observed up to 150 K. In the work of Demidov *et al.* [82], Mn-doped Si films were prepared on Al_2_O_3_ substrates by laser deposition. They observed anomalous Hall effect even up to room temperature. However, in both papers there is no correlation between Hall effect and magnetization.

## 4. Summary and outlook

The research on diluted magnetic semiconductors has been intensified in the last 30 years. The major activities were focused on the GaAs:Mn system and on the transition metal doped wide bandgap semiconductors GaN:Mn, ZnO:Mn, and ZnO:Co. However, due to the intrinsic n-type conductivity of ZnO, the later case is the most questionable research topic. Recent systematic investigations on transition metal doped ZnO hint towards that the observed ferromagnetism mostly comes from secondary phases [83], from a canted antiparallel alignment of magnetic moments in ZnO:Mn [84] or from acceptor-like defects [85]. The diluted Co ions in ZnO:Co are purely paramagnetic [86], and the lack of electron-mediated ferromagnetism can be construed. In Table 2, we show the possible states of Mn impurities inside different semiconductors. Obviously, the formation of interstitial Mn ions must be suppressed, and co-doping with shallow acceptors can be considered. For example, co-doping with shallow acceptors has been proved helpful in increasing the hole concentration and Curie temperature in GaAs:Mn films with a small Mn content [87] and in ZnTe:Mn [47].

**Table 2:** The properties of substitutional and interstitial Mn impurity inside different semiconductors. Possible shallow acceptor co-dopants are also listed. Detailed information has been collected in the database of “New Semiconductor Materials. Characteristics and Properties” at http://www.ioffe.ru/SVA/NSM/.

	II-VI		III-V		Elemental	
	ZnO	ZnTe	GaAs	GaN	Ge	Si
Substitutional Mn	isovalent	isovalent	acceptor	acceptor	double acceptor	-
Interstitial Mn	-	-	donor	donor	donor	donor or acceptor
Acceptor co-dopants	P, N	N	Be, Mg	Zn	Ga	B, Al, Ga

The investigation on Ge:Mn and Si:Mn systems is relatively rare, since it was believed that transition metal ions in Si are fast interstitial diffusers even at low temperatures. From this review on the experimental status of Mn-doped Ge and Si, we can draw some, not conclusive but preliminary remarks.

For Mn-doped Ge:

- There is a considerable amount of Mn diluted inside the Ge matrix, resulting in p-type doping.
- Mn ions inside the Ge matrix tend to form secondary phases or Mn-rich nanostructures even at preparation temperatures as low as 70 °C.
- The observed ferromagnetism in Mn-doped Ge is usually multi-fold originated. The magnetic phase ordering below around 20 K likely results from the hole-mediated coupling between diluted Mn ions.
- The previously reported magnetotransport properties are much different from those of GaAs:Mn, and cannot be taken as the evidence of carrier-mediated ferromagnetism.
- Besides LT-MBE, an alternative preparation approach is ion implantation followed by pulsed laser annealing offering a high temperature (at the Ge molten point) and short time (as short as nano-second) process. It promises a more effective substitution of Mn inside the Ge matrix.

For Mn-doped Si:

- It is still unclear if Mn can substitute the Si site.
- The observed ferromagnetism in Mn-doped Si is usually multi-fold originated. One source is nanocrystalline MnSi_1.7_.
- It is difficult to make comments on the existence of carrier mediated ferromagnetism in Mn-doped Si since there are only limited reports on its magnetotransport properties.
- One possible approach for a Si FMS would be ion implantation of other 3*d* transition metals followed by pulsed laser annealing. The chosen 3*d* transition metal ions should quench out of interstitial sites during pulsed laser annealing and form acceptor states in Si.

## References

[1] Binasch G., Grünberg P., Saurenbach F., Zinn W. (1989). Enhanced magnetoresistance in layered magnetic structures with antiferromagnetic interlayer exchange. Phys. Rev. B.

[2] Baibich M.N., Broto J.M., Fert A., van Dau F.N., Petroff F., Eitenne P., Creuzet G., Friederich A., Chazelas J. (1988). Giant Magnetoresistance of (001)Fe/(001)Cr Magnetic Superlattices. Phys. Rev. Lett..

[3] Datta S., Das B. (1990). Electronic analog of the electrooptic modulator. Appl. Phys. Lett..

[4] Schmidt G., Ferrand D., Molenkamp L.W., Filip A.T., van Wees B.J. (2000). Fundamental obstacle for electrical spin injection from a ferromagnetic metal into a diffusive semiconductor. Phys. Rev. B.

[5] Jungwirth T., Wang K.Y., Masek J., Edmonds K.W., Konig J., Sinova J., Polini M., Goncharuk N.A., MacDonald A.H., Sawicki M., Rushforth A.W., Campion R.P., Zhao L.X., Foxon C.T., Gallagher B.L. (2005). Prospects for high temperature ferromagnetism in (Ga,Mn)As semiconductors. Phys. Rev. B.

[6] Ohno Y., Young D.K., Beschoten B., Matsukura F., Ohno H., Awschalom D.D. (1999). Electrical spin injection in a ferromagnetic semiconductor heterostructure. Nature.

[7] Ohno H., Chiba D., Matsukura F., Omiya T., Abe E., Dietl T., Ohno Y., Ohtani K. (2000). Electric-field control of ferromagnetism. Nature.

[8] Tanaka M., Higo Y. (2001). Large Tunneling Magnetoresistance in GaMnAs /AlAs /GaMnAs Ferromagnetic Semiconductor Tunnel Junctions. Phys. Rev. Lett..

[9] Humpfner S., Pappert K., Wenisch J., Brunner K., Gould C., Schmidt G., Molenkamp L.W., Sawicki M., Dietl T. (2007). Lithographic engineering of anisotropies in (Ga,Mn)As. Appl. Phys. Lett..

[10] Dietl T., Ohno H., Matsukura F., Cibert J., Ferrand D. (2000). Zener model description of ferromagnetism in zinc-blende magnetic semiconductors. Science.

[11] Sapega V.F., Moreno M., Ramsteiner M., Däweritz L., Ploog K.H. (2005). Polarization of Valence Band Holes in the (Ga,Mn)As Diluted Magnetic Semiconductor. Phys. Rev. Lett..

[12] Neumaier D., Wagner K., Geißler S., Wurstbauer U., Sadowski J., Wegscheider W., Weiss D. (2007). Weak Localization in Ferromagnetic (Ga,Mn)As Nanostructures. Phys. Rev. Lett..

[13] Tang H.X., Kawakami R.K., Awschalom D.D., Roukes M.L. (2003). Giant Planar Hall Effect in Epitaxial (Ga,Mn)As Devices. Phys. Rev. Lett..

[14] Ciorga M., Schlapps M., Einwanger A., Geiler S., Sadowski J., Wegscheider W., Weiss D. (2007). TAMR effect in (Ga,Mn)As-based tunnel structures. New J. Phys..

[15] Jungwirth T., Niu Q., MacDonald A.H. (2002). Anomalous Hall Effect in Ferromagnetic Semiconductors. Phys. Rev. Lett..

[16] Burch K.S., Shrekenhamer D.B., Singley E.J., Stephens J., Sheu B.L., Kawakami R.K., Schiffer P., Samarth N., Awschalom D.D., Basov D.N. (2006). Impurity Band Conduction in a High Temperature Ferromagnetic Semiconductor. Phys. Rev. Lett..

[17] Wu H., Kratzer P., Scheffler M. (2005). First-principles study of thin magnetic transition-metal silicide films on Si(001). Phys. Rev. B.

[18] Li W., Kang Q., Lin Z., Chu W., Chen D., Wu Z., Yan Y., Chen D., Huang F. (2006). Paramagnetic anisotropy of Co-doped ZnO single crystal. Appl. Phys. Lett..

[19] Woodbury H.H., Tyler W.W. (1955). Properties of Germanium Doped with Manganese. Phys. Rev..

[20] Park Y.D., Hanbicki A.T., Erwin S.C., Hellberg C.S., Sullivan J.M., Mattson J.E., Ambrose T.F., Wilson A., Spanos G., Jonker B.T. (2002). A Group-IV Ferromagnetic Semiconductor: Mn*_x_*Ge_1_*_−x_*. Science.

[21] Li A.P., Zeng C., van Benthem K., Chisholm M.F., Shen J., Rao S.V.S.N., Dixit S.K., Feldman L.C., Petukhov A.G., Foygel M., Weitering H.H. (2007). Dopant segregation and giant magnetoresistance in manganese-doped germanium. Phys. Rev. B.

[22] Tsui F., He L., Ma L., Tkachuk A., Chu Y.S., Nakajima K., Chikyow T. (2003). Novel Germanium-Based Magnetic Semiconductors. Phys. Rev. Lett..

[23] Jamet M., Barski A., Devillers T., Poydenot V., Dujardin R., Bayle-Guillemaud P., Rothman J., Bellet-Amalric E., Marty A., Cibert J., Mattana R., Tatarenko S. (2006). High-Curie-temperature ferromagnetism in self-organized Ge_1_*_−x_*Mn*_x_* nanocolumns. Nat. Mater..

[24] Ahlers S., Bougeard D., Riedl H., Abstreiter G., Trampert A., Kipferl W., Sperl M., Bergmaier A., Dollinger G. (2006). Ferromagnetic Ge(Mn) nanostructures. Physica E.

[25] Pinto N., Morresi L., Ficcadenti M., Murri R., D‘Orazio F., Lucari F., Boarino L., Amato G. (2005). Magnetic and electronic transport percolation in epitaxial Ge_1_*_−x_*Mn*_x_* films. Phys. Rev. B.

[26] Kang J.S., Kim G., Wi S.C., Lee S.S., Choi S., Cho S., Han S.W., Kim K.H., Song H.J., Shin H.J., Sekiyama A., Kasai S., Suga S., Min B.I. (2005). Spatial Chemical Inhomogeneity and Local Electronic Structure of Mn-Doped Ge Ferromagnetic Semiconductors. Phys. Rev. Lett..

[27] Picozzi S., Ottaviano L., Passacantando M., Profeta G., Continenza A., Priolo F., Kim M., Freeman A.J. (2005). X-ray absorption spectroscopy in Mn*_x_*Ge_1_*_−x_* diluted magnetic semiconductor: Experiment and theory. Appl. Phys. Lett..

[28] Gambardella P., Claude L., Rusponi S., Franke K.J., Brune H., Raabe J., Nolting F., Bencok P., Hanbicki A.T., Jonker B.T., Grazioli C., Veronese M., Carbone C. (2007). Surface characterization of Mn*_x_*Ge_1_*_−x_* and Cr*_y_*Mn*_x_*Ge_1_*_−x−y_* dilute magnetic semiconductors. Phys. Rev. B.

[29] Biegger E., Stäheli L., Fonin M., Rüdiger U., Dedkov Y.S. (2007). Intrinsic ferromagnetism versus phase segregation in Mn-doped Ge. J. Appl. Phys..

[30] Ahlers S., Stone P.R., Sircar N., Arenholz E., Dubon O.D., Bougeard D. (2009). Comparison of the magnetic properties of GeMn thin films through Mn L-edge x-ray absorption. Appl. Phys. Lett..

[31] Bihler C., Jaeger C., Vallaitis T., Gjukic M., Brandt M.S., Pippel E., Woltersdorf J., Gösele U. (2006). Structural and magnetic properties of Mn_5_Ge_3_ clusters in a dilute magnetic germanium matrix. Appl. Phys. Lett..

[32] Passacantando M., Ottaviano L., D’Orazio F., Lucari F., Biase M.D., Impellizzeri G., Priolo F. (2006). Growth of ferromagnetic nanoparticles in a diluted magnetic semiconductor obtained by Mn^+^ implantation on Ge single crystals. Phys. Rev. B.

[33] Wang Y., Zou J., Zhao Z., Han X., Zhou X., Wang K.L. (2008). Direct structural evidences of Mn_11_Ge_8_ and Mn_5_Ge_2_ clusters in Ge_0.96_Mn_0.04_ thin films. Appl. Phys. Lett..

[34] Bougeard D., Ahlers S., Trampert A., Sircar N., Abstreiter G. (2006). Clustering in a Precipitate-Free GeMn Magnetic Semiconductor. Phys. Rev. Lett..

[35] Devillers T., Jamet M., Barski A., Poydenot V., Bayle-Guillemaud P., Bellet-Amalric E., Cherifi S., Cibert J. (2007). Structure and magnetism of self-organized *Ge*_1_*_−x_Mn_x_* nanocolumns on *Ge*(001). Phys. Rev. B.

[36] Yu I.S., Jamet M., Devillers T., Barski A., Bayle-Guillemaud P., Beigné C., Rothman J., Baltz V., Cibert J. (2010). Spinodal decomposition to control magnetotransport in (Ge,Mn) films. Phys. Rev. B.

[37] Tardif S., Favre-Nicolin V., Lanc¸on F., Arras E., Jamet M., Barski A., Porret C., Bayle-Guillemaud P., Pochet P., Devillers T., Rovezzi M. (2010). Strain and correlation of self-organized *Ge*_1_*_−x_Mn_x_* nanocolumns embedded in Ge (001). Phys. Rev. B.

[38] Tardif S., Cherifi S., Jamet M., Devillers T., Barski A., Schmitz D., Darowski N., Thakur P., Cezar J.C., Brookes N.B., Mattana R., Cibert J. (2010). Exchange bias in GeMn nanocolumns: The role of surface oxidation. Appl. Phys. Lett..

[39] Jaeger C., Bihler C., Vallaitis T., Goennenwein S.T.B., Opel M., Gross R., Brandt M.S. (2006). Spin-glass-like behavior of Ge:Mn. Phys. Rev. B.

[40] Li A.P., Shen J., Thompson J.R., Weitering H.H. (2005). Ferromagnetic percolation in Mn*_x_*Ge_1_*_−x_* dilute magnetic semiconductor. Appl. Phys. Lett..

[41] Morgunov R.B., Dmitriev A.I., Kazakova O.L. (2009). Percolation ferromagnetism and spin waves in Ge:Mn thin films. Phys. Rev. B.

[42] Zhou S., Shalimov A., Potzger K., Jeutter N.M., Baehtz C., Helm M., Fassbender J., Schmidt H. (2009). Memory effect of Mn_5_Ge_3_ nanomagnets embedded inside a Mn-diluted Ge matrix. Appl. Phys. Lett..

[43] Wang W.Z., Deng J.J., Lu J., Sun B.Q., Zhao J.H. (2007). Memory effect in a system of zincblende Mn-rich Mn(Ga)As nanoclusters embedded in GaAs. Appl. Phys. Lett..

[44] Sun Y., Salamon M.B., Garnier K., Averback R.S. (2003). Memory Effects in an Interacting Magnetic Nanoparticle System. Phys. Rev. Lett..

[45] Hayashi T., Tanaka M., Nishinaga T., Shimada H. (1997). Magnetic and magnetotransport properties of new III-V diluted magnetic semiconductors: GaMnAs. J. Appl. Phys..

[46] Ohno H., Munekata H., Penney T., von Molnár S., Chang L.L. (1992). Magnetotransport properties of p-type (In,Mn)As diluted magnetic III-V semiconductors. Phys. Rev. Lett..

[47] Ferrand D., Cibert J., Wasiela A., Bourgognon C., Tatarenko S., Fishman G., Andrearczyk T., Jaroszyński J., Koleśnik S., Dietl T., Barbara B., Dufeu D. (2001). Carrier-induced ferromagnetism in p-Zn_1_*_−x_*Mn*_x_*Te. Phys. Rev. B.

[48] Ohno H., Shen A., Matsukura F., Oiwa A., Endo A., Katsumoto S., Iye Y. (1996). (Ga,Mn)As: A new diluted magnetic semiconductor based on GaAs. Appl. Phys. Lett..

[49] Li A.P., Wendelken J.F., Shen J., Feldman L.C., Thompson J.R., Weitering H.H. (2005). Magnetism in Mn*_x_*Ge_1_*_−x_* semiconductors mediated by impurity band carriers. Phys. Rev. B.

[50] Riss O., Gerber A., Korenblit I.Y., Suslov A., Passacantando M., Ottaviano L. (2009). Magnetization-driven metal-insulator transition in strongly disordered Ge:Mn magnetic semiconductors. Phys. Rev. B.

[51] Zeng C., Zhang Z., van Benthem K., Chisholm M.F., Weitering H.H. (2008). Optimal Doping Control of Magnetic Semiconductors via Subsurfactant Epitaxy. Phys. Rev. Lett..

[52] Gareev R.R., Bugoslavsky Y.V., Schreiber R., Paul A., Sperl M., Döppe M. (2006). Carrier-induced ferromagnetism in Ge(Mn,Fe) magnetic semiconductor thin-film structures. Appl. Phys. Lett..

[53] Deng J.X., Tian Y.F., He S.M., Bai H.L., Xu T.S., Yan S.S., Dai Y.Y., Chen Y.X., Liu G.L., Mei L.M. (2009). Strong anisotropy of magnetization and sign reversion of ordinary Hall coefficient in single crystal Ge_1_*_−x_*Mn*_x_* magnetic semiconductor films. Appl. Phys. Lett..

[54] Yamamoto Y., Itaya S., Suga K., Takenobu T., Iwasa Y., Hagiwara M., Kindo K., Hori H. (2006). Anomalous Hall- and magneto-resistances on Cr-doped Ge in high magnetic fields observed up to room temperature. J. Phys.: Conf. Ser..

[55] Zhou S., Bürger D., Helm M., Schmidt H. (2009). Anomalous Hall effect in Ge:Mn with low Mn concentrations. Appl. Phys. Lett..

[56] Look D.C., Walters D.C., Manasreh M.O., Sizelove J.R., Stutz C.E., Evans K.R. (1990). Anomalous Hall-effect results in low-temperature molecular-beam-epitaxial GaAs: Hopping in a dense EL2-like band. Phys. Rev. B.

[57] Watts S.M., Wirth S., von Molnár S., Barry A., Coey J.M.D. (2000). Evidence for two-band magnetotransport in half-metallic chromium dioxide. Phys. Rev. B.

[58] Jung D.W., Noh J.P., Islam A.Z.M.T., Otsuka N. (2008). Large anomalous Hall resistance of pair delta-doped GaAs structures grown by molecular-beam epitaxy. J. Appl. Phys..

[59] White C.W., Wilson S.R., Appleton B.R., F. W. Young J. (1980). Supersaturated substitutional alloys formed by ion implantation and pulsed laser annealing of group-III and group-V dopants in silicon. J. Appl. Phys..

[60] Zhou S., Bürger D., Mücklich A., Baumgart C., Skorupa W., Timm C., Oesterlin P., Helm M., Schmidt H. (2010). Hysteresis in the magnetotransport of manganese-doped germanium: Evidence for carrier-mediated ferromagnetism. Phys. Rev. B.

[61] Yuldashev S.U., Jeon H.C., Im H.S., Kang T.W., Lee S.H., Furdyna J.K. (2004). Anomalous Hall effect in insulating Ga_1_*_−x_*Mn*_x_* As. Phys. Rev. B.

[62] Scarpulla M.A., Cardozo B.L., Farshchi R., Oo W.M.H., McCluskey M.D., Yu K.M., Dubon O.D. (2005). Ferromagnetism in *Ga*_1_*_−x_Mn_x_P*: Evidence for Inter-Mn Exchange Mediated by Localized Holes within a Detached Impurity Band. Phys. Rev. Lett..

[63] Zhou S., Bürger D., Skorupa W., Oesterlin P., Helm M., Schmidt H. (2010). The importance of hole concentration in establishing carrier-mediated ferromagnetism in Mn-doped Ge. Appl. Phys. Lett..

[64] Weber E. (1983). Transition Metals in Silicon. Appl. Phys. A.

[65] Wu H., Kratzer P., Scheffler M. (2007). Density-Functional Theory Study of Half-Metallic Heterostructures: Interstitial Mn in Si. Phys. Rev. Lett..

[66] Zhang F.M., Liu X.C., Gao J., Wu X.S., Du Y.W., Zhu H., Xiao J.Q., Chen P. (2004). Investigation on the magnetic and electrical properties of crystalline Mn_0.05_Si_0.95_ films. Appl. Phys. Lett..

[67] Bolduc M., Awo-Affouda C., Stollenwerk A., Huang M.B., Ramos F.G., Agnello G., LaBella V.P. (2005). Above room temperature ferromagnetism in Mn-ion implanted Si. Phys. Rev. B.

[68] Zhou S., Potzger K., Zhang G., Mücklich A., Eichhorn F., Schell N., Grötzschel R., Schmidt B., Skorupa W., Helm M., Fassbender J., Geiger D. (2007). Structural and magnetic properties of Mn-implanted Si. Phys. Rev. B.

[69] Ko V., Teo K.L., Liew T., Chong T.C., MacKenzie M., MacLaren I., Chapman J.N. (2008). Origins of ferromagnetism in transition-metal doped Si. J. Appl. Phys..

[70] Yabuuchi S., Kageshima H., Ono Y., Nagase M., Fujiwara A., Ohta E. (2008). Origin of ferromagnetism of MnSi_1.7_ nanoparticles in Si: First-principles calculations. Phys. Rev. B.

[71] Yabuuchi S., Ono Y., Nagase M., Kageshima H., Fujiwara A., Ohta E. (2008). Ferromagnetism of Manganese–Silicide Nanopariticles in Silicon. J. J. Appl. Phys..

[72] Wolska A., Lawniczak-Jablonska K., Klepka M., Walczak M.S., Misiuk A. (2007). Local structure around Mn atoms in Si crystals implanted with Mn^+^ studied using X-ray absorption spectroscopy techniques. Phys. Rev. B.

[73] Ye J., Jiang Y., Liu Q., Yao T., Pan Z., Oyanagi H., Sun Z., Yan W., Wei S. (2009). Cosputtered Mn-doped Si thin films studied by x-ray spectroscopy. J. Appl. Phys..

[74] Ko V., Teo K.L., Liew T., Chong T.C., Liu T., Wee A.T.S., Du A.Y., Stoffel M., Schmidt O.G. (2008). Correlation of structural and magnetic properties of ferromagnetic Mn-implanted Si_1_*_−x_*Ge*_x_* films. J. Appl. Phys..

[75] Awo-Affouda C., Bolduc M., Huang M.B., Ramos F., Dunn K.A., Thiel B., Agnello G., LaBella V.P. (2006). Observation of crystallite formation in ferromagnetic Mn-implanted Si. J. Vac. Sci. Technol. A.

[76] Zou Z.Q., Wang H., Wang D., Wang Q.K., Mao J.J., Kong X.Y. (2007). Epitaxial growth of manganese silicide nanowires on Si(111)-7 × 7 surfaces. Appl. Phys. Lett..

[77] Wang D., Zou Z.Q. (2009). Formation of manganese silicide nanowires on Si(111) surfaces by the reactive epitaxy method. Nanotechnology.

[78] Yamamoto Y., Itaya S., Suga K., Takenobu T., Iwasa Y., Hagiwara M., Kindo K., Hori H. (1935). Anomalous Hall- and magneto- resistances on Cr-doped Ge in high magnetic fields observed up to room temperature. Z. Phys..

[79] Shalimov A., Potzger K., Geiger D., Lichte H., Talut G., Misiuk A., Reuther H., Stromberg F., Zhou S., Baehtz C., Fassbender J. (2009). Fe nanoparticles embedded in MgO crystals. J. Appl. Phys..

[80] Zhou S., Shalimov A., Potzger K., Helm M., Fassbender J., Schmidt H. (2009). MnSi_1.7_ nanoparticles embedded in Si: Superparamagnetism with collective behavior. Phys. Rev. B.

[81] Yao J.H., Li S.C., Lan M.D., Chin T.S. (2009). Mn-doped amorphous Si:H films with anomalous Hall effect up to 150 K. Appl. Phys. Lett..

[82] Demidov E., Aronzon B., Gusev S., Karzanov V., Lagutin A., Lesnikov V., Levchuk S., Nikolaev S., Perov N., Podolskii V., Rylkov V., Sapozhnikov M., Lashkul A. (2009). High-temperature ferromagnetism in laser-deposited layers of silicon and germanium doped with manganese or iron impurities. J. Magn. Magn. Mater..

[83] Zhou S., Potzger K., von Borany J., Grötzschel R., Skorupa W., Helm M., Fassbender J. (2008). Crystallographically oriented Co and Ni nanocrystals inside ZnO formed by ion implantation and postannealing. Phys. Rev. B.

[84] Diaconu M., Schmidt H., Hochmuth H., Lorenz M., Benndorf G., Spemann D., Setzer A., Esquinazi P., Pppl A., von Wenckstern H., Nielsen K.W., Gross R., Schmid H., Mader W., Wagner G., Grundmann M. (2006). Room-temperature ferromagnetic Mn-alloyed ZnO films obtained by pulsed laser deposition. J. Magn. Magn. Mater..

[85] Xu Q., Schmidt H., Zhou S., Potzger K., Helm M., Hochmuth H., Lorenz M., Setzer A., Esquinazi P., Meinecke C., Grundmann M. (2008). Room temperature ferromagnetism in ZnO films due to defects. Appl. Phys. Lett..

[86] Xu Q., Zhou S., Marko D., Potzger K., Fassbender J., Vinnichenko M., Helm M., Hochmuth H., Lorenz M., Grundmann M., Schmidt H. (2009). Paramagnetism in Co-doped ZnO films. J. Phys. D: Appl. Phys..

[87] Lee S., Chung S.J., Choi I.S., Yuldeshev S.U., Im H., Kang T.W., Lim W.L., Sasaki Y., Liu X., Wojtowicz T., Furdyna J.K. (2003). Effect of Be doping on the properties of GaMnAs ferromagnetic semiconductors. J. Appl. Phys..

[88] Özer M. (2010). Handbook of Spintronic Semiconductors.

